# Androgen Deprivation Therapy in High-Risk Localized and Locally Advanced Prostate Cancer

**DOI:** 10.3390/cancers14071803

**Published:** 2022-04-01

**Authors:** Hiroaki Iwamoto, Kouji Izumi, Tomoyuki Makino, Atsushi Mizokami

**Affiliations:** 1 Department of Integrative Cancer Therapy and Urology, Kanazawa University Graduate School of Medical Science, 13-1 Takara-Machi, Kanazawa 920-8640, Ishikawa, Japan; iwamoto-h@med.kanazawa-u.ac.jp (H.I.); mackeeen511@gmail.com (T.M.); mizokami@staff.kanazawa-u.ac.jp (A.M.); 2 Department of Urology, Ishikawa Prefectural Central Hospital, Kanazawa 920-8530, Ishikawa, Japan

**Keywords:** prostate cancer, androgen deprivation therapy, high-risk, localized, locally advanced

## Abstract

**Simple Summary:**

Androgen deprivation therapy alone is commonly performed for metastatic prostate cancer but is generally not recommended for the treatment of high-risk localized or locally advanced prostate cancer. In this article, we will discuss the position, indications, and future possibilities of ADT for high-risk localized or locally advanced prostate cancer.

**Abstract:**

The recommended treatment for high-risk localized or locally advanced prostate cancer is radical prostatectomy plus extended pelvic lymph node dissection or radiation therapy plus long-term androgen deprivation therapy. However, some patients are treated with androgen deprivation therapy alone for various reasons. In this review, we will discuss the position, indications, complications, and future prospects of androgen deprivation therapy for high-risk localized and locally advanced prostate cancer.

## 1. Introduction

Prostate cancer (PC) is the most common cancer in men and the leading cause of cancer-related deaths in developed countries [1,2]. Most PC deaths are caused by metastatic disease [3]. Approximately 10% of new PC cases are diagnosed with distant metastasis [4,5,6]. The development and dissemination of prostate-specific antigen (PSA) screening have contributed to early PC detection, which in turn has reduced PC-related mortality [7,8,9,10]. However, approximately 15% of newly diagnosed PCs are high-risk PCs [11]. While localized PC generally has a good prognosis, high-risk PC has a significantly worse prognosis than low- and intermediate-risk PC, with a 15-year PC-specific mortality rate of 22–38% [11,12,13]. Guidelines differ slightly in their definition of high-risk PC, including locally advanced PC (Table 1). In the D’Amico risk classification, the European Association of Urology (EAU) guidelines, and European Society for Medical Oncology (ESMO) guidelines, patients are classified as high-risk if they meet clinical stage T2c or a PSA level of ≥20 ng/mL or Gleason score of 8–10, while the National Comprehensive Cancer Network (NCCN) guidelines classify patients as high-risk at clinical stage T3 or higher [14,15,16,17]. The EAU guidelines classify locally advanced PC at clinical stage T3 or higher or clinical stage N1, and the NCCN guidelines classify very high-risk at clinical stage T3b or higher or primary Gleason pattern 5 or >4 cores with grade group 4 or 5 [15,16]. Briefly, patients with clinical stage T2c, a PSA level of ≥20 ng/mL, or a Gleason score of 8–10 are considered to have high-risk PC. Although each guideline differs slightly, radical prostatectomy (RP) + extended pelvic lymph node dissection (ePLND) or radiation therapy (RT) + long-term androgen deprivation therapy (ADT) is recommended for the treatment of high-risk or locally advanced PC, and ADT alone is currently indicated in a few cases [15,16,17]. However, ADT has been used aggressively for localized PC in Japan. According to data from 10,280 PC patients diagnosed in Japan in 2004, 41% of non-metastatic castration-sensitive prostate cancer (nmCSPC) patients received ADT as initial treatment [5]. In this review, we provide an overview of ADT therapy, including the position and future possibilities of ADT alone in high-risk or locally advanced PC. Herein, ADT is defined as including gonadotropin-releasing hormone (GnRH) agonist alone, GnRH agonist alone, anti-androgenic agent alone, and combined androgen blockade (CAB) therapy.

## 2. ADT

### 2.1. History of ADT

After Huggins and Hodges reported the efficacy of castration therapy, ADT became the gold standard for advanced PC [18]. Surgical castration had been the only method of castration, but Schally and Guillemin elucidated the structure of GnRH, which led to the development of GnRH agonists, and medical castration became possible [19]. Studies have reported the efficacy and tolerability of GnRH agonists as a first-line treatment for advanced PC and concluded that the survival rate, disease progression, and time to treatment failure are comparable between GnRH agonist therapy and orchiectomy [20,21,22,23]. Surgical castration is a simple and cost-effective outpatient procedure, while the advantage of medical castration is the avoidance of surgery [20,21,22,23,24]. In recent years, the rate of surgical castration has been reported to be less than 9% [24]. Although castration reduces serum testosterone levels by approximately 90%, 20–40% of dihydrotestosterone (DHT) remains in human PC tissue [25,26,27]. As this residual androgen may cause inadequate treatment of PC or relapse, CAB therapy, which combines ADT with an antiandrogen drug, has been proposed [28,29,30]. Recently, GnRH antagonists are developed. GnRH agonists have been reported to result in a transient increase in testosterone levels that occurs early in the administration, which is called a testosterone surge that can cause urinary tract obstruction and spinal cord compression [31,32,33]. GnRH antagonists do not cause testosterone surges, and testosterone levels reach castration levels early, so they may be particularly useful in patients with symptomatic metastatic PC [34]. In addition, ADT has made progress with the development of the new androgen receptor signaling-targeted agent (ARSTs), such as enzalutamide, abiraterone acetate, apalutamide, and darolutamide [35,36,37,38]. The effects of ADT are generally not permanent and eventually lead to castration-resistant PC (CRPC) [6]. The treatment for CRPC includes alternative ADT [39], ARST [35,36,37,38], and chemotherapy such as docetaxel [40,41] and cabazitaxel [42,43].

ADT is effective in improving patients’ quality of life by reducing bone pain, pathological fractures, spinal cord compression, and ureteral obstruction, which are symptoms specific to advanced PC, such as bone metastases or local enlargement of PC [18,44,45]. However, ADT has various adverse effects, such as hot flashes [46,47,48,49,50], sexual dysfunction [46,49,50,51,52], breast enlargement [48,49,53,54], depression [49,55], dementia [56,57,58,59,60,61], osteoporosis [62,63,64,65,66], obesity [46,67], diabetes [68,69,70,71,72,73], and cardiovascular (CV) toxicity [68,74,75,76,77,78,79,80].

Dementia, osteoporosis, and CV toxicity are important side effects of ADT in older patients. We summarize these AEs for older patients with PC in the next section.

### 2.2. ADT for High-Risk Localized and Locally Advanced PC

Various guidelines do not recommend ADT alone as an initial treatment for high-risk or locally advanced PC [15,16,17]. However, in clinical practice, some patients, especially older ones, are treated with ADT alone as the initial therapy. In the USA, the use of GnRH agonists has increased since the 1990s [81], and as of 2009, 22% of patients with localized PC aged >66 years were being treated with ADT alone [82]. In Japan, approximately 30% of patients with localized PC were treated with ADT alone [5]. As shown in Table 2 and Table 3, many studies have reported on the efficacy of ADT alone for localized PC, but worldwide, there are more negative reports.

#### 2.2.1. Negative Data of ADT for High-Risk Localized and Locally Advanced PC

A cohort study of 844 patients with localized PC who underwent total prostatectomy, RT, watchful waiting, ADT, or other treatment, with data collected from the Geneva Cancer Registry, revealed that patients who received hormone therapy alone had increased PC-specific mortality at 5 years [83].

A retrospective cohort study of 340 patients diagnosed with localized PC and followed up with ADT or no treatment at a single center in Singapore found no improvement in 5-year all-cause mortality or PC-specific mortality (PCSM) when ADT was initiated within 12 months of diagnosis [84].

A retrospective cohort study comparing 7867 patients who were newly diagnosed with localized PC and received ADT with 11,404 patients who did not receive ADT was selected from the population-based Surveillance, Epidemiology, and End Results (SEER) program database and linked Medicare files. The study showed that ADT was not associated with improved survival for the majority of older men compared with conservative management [85].

A retrospective study of 15,170 patients with newly diagnosed clinically localized PC who were not receiving curative treatment was conducted using data from three integrated healthcare delivery systems within the HMO Cancer Research Network in the USA. The results showed that ADT was not associated with either overall or PCSM risk. However, ADT predominantly reduced the risk of all-cause mortality only in a subgroup of men at high-risk for cancer progression [86].

A retrospective cohort study of 66,717 patients aged ≥66 years with localized PC who did not receive curative treatment, from the National Cancer Institute’s SEER program and Medicare data, found that primary ADT was not associated with improved long-term overall survival (OS) or disease-specific survival at 15 years [87].

In a retrospective cohort study of 46,376 patients newly diagnosed with locally advanced or localized PC from the National Cancer Institute’s SEER program and Medicare data and not treated with curative intent, ADT was associated with decreased survival compared with observation management [82].

As shown above, many large cohort studies have rejected the efficacy of ADT for localized PC. However, some studies have shown the efficacy of ADT in localized PC.

#### 2.2.2. Positive Data of ADT for High-Risk Localized and Locally Advanced PC

In a previous study, 57 patients with newly diagnosed locally advanced or localized PC who discontinued long-term CAB therapy were followed for at least 5 years. Among 20 patients with stage T2 to T3 cancer who discontinued continuous CAB therapy after 6.5 years, two cases of PSA elevations occurred, with a 90% non-failure rate. This study suggested that long-term and continuous CAB was associated with the possibility of long-term control or cure of localized PC [88].

In a prospective cohort study of 151 patients with newly diagnosed locally advanced or localized PC who underwent ADT from 104 sites in Japan, ADT reduced PC progression, resulting in a life expectancy similar to that of the normal population [89].

A retrospective study of data from The Cancer of the Prostate Strategic Urologic Research Endeavor (CaPSURE) in the USA compared 993 patients with newly diagnosed localized PC who received ADT with 6051 patients who did not receive ADT. The results revealed that ADT use controlled the disease in the majority of patients with PC at an intermediate period of 5 years [90].

In an analysis of the Japan Study Group of Prostate Cancer (J-CaP) surveillance study of 15,461 patients with locally advanced or localized PC in Japan, ADT resulted in long-term survival rates similar to that in the general population [91].

In a report of 410 patients with intermediate- to high-risk localized PC treated with ADT alone from five centers in Japan, the expected survival rate was similar to that of the general population in the absence of capsular invasion and with a Gleason score of ≥8 [92].

In the European Organization for Research and Treatment of Cancer (EORTC) trial 30891, a randomized, prospective study compared 492 patients with PC without distant metastases who received immediate ADT with 493 patients who received delayed ADT. The results indicated that the delayed ADT group had a significantly inferior OS rate to the immediate ADT group [93].

In a pooled analysis of a randomized trial of 4141 patients with unfavorable-risk PC, ADT use was associated with a decreased risk of PCSM and all-cause mortality [80].

As described above, most of the studies demonstrating the efficacy of ADT for localized PC were reported from Japan. However, there have been reports of racial differences in the efficacy of ADT for localized PC.

#### 2.2.3. Differences in the Efficacy of ADT by Race

A retrospective study of 165 patients with PC who underwent ADT at The Queen’s Medical Center in Honolulu compared 59 Caucasian men (CM) and 105 Japanese American men (JAM) [94]. Although no significant difference was found in the patient background, JAMs who received ADT had a better prognosis than CMs in terms of both overall and cause-specific survival (*p* = 0.001 and 0.036, respectively). The multivariate analysis also revealed that race was one of the significant prognostic factors (*p* = 0.03).

A retrospective study compared data from a total of 15,513 patients with PC who received ADT from the CaPSURE database in the USA and the J-CaP database in Japan [95]. Men who underwent ADT at J-CaP (*n* = 13,880) were older and had higher risk of disease than men who underwent ADT at CaPSURE (*n* = 1633) and had a higher rate of CAB (66.9% vs. 46.4%). The multivariate regression showed that the sub-hazard ratio for PCSM was 0.52 (95% confidence interval 0.40–0.68) for J-CaP versus CaPSURE, and the adjusted PCSM for men receiving ADT in Japan was less than half that of men in the USA.

Although further large-scale prospective studies are awaited, Asians, including Japanese, may be expected to benefit more from ADT than Caucasians.

#### 2.2.4. Position of ADT in High-Risk Localized and Locally Advanced PC

The efficacy of RP and RT for localized PC has been recognized [96,97,98]. A randomized clinical trial (RCT) reported that improved survival with long-term ADT plus RT for patients with locally advanced PC has led to the recommendation of combined RT and ADT for high-risk cases [99,100]. RP was not recommended for patients with high-risk PC; however, recent reports have led to a reevaluation. In a meta-analysis including 118,830 patients and comparing the prognosis of RP and RT for localized PC, the prognosis was significantly better with RP, even in the high-risk group [101]. In a retrospective study of 42,765 patients with high-risk PC, the RP group had a significantly better prognosis than the RT plus ADT group [102]. Based on the above, RP plus ePLND or RT plus ADT is recommended for the treatment of high-risk or locally advanced PC [15,16,17]. However, RP is not recommended for patients with an expected life expectancy of ≤5 years, and ADT alone may be an option [15]. Older patients are more likely to have several comorbidities and should be aware of adverse events (AEs) from ADT.

### 2.3. Evidence of ARST for Castration-Sensitive Prostate Cancer

In recent years, evidence of ARST for castration-sensitive prostate cancer (CSPC) has been accumulating. In the ARCHES trial, in which 1150 patients with metastatic CSPC (mCSPC) were randomized 1:1 to enzalutamide plus ADT or placebo plus ADT, the enzalutamide plus ADT group had significantly longer radiographic progression-free survival than the placebo group (not reached vs. 19.0 months, *p* < 0.001, HR = 0.39) [103]. In the ENZAMET study, in which 1125 patients with mCSPC were randomized to enzalutamide plus ADT or non-steroidal antiandrogen plus ADT, both groups did not reach the median OS; however, the enzalutamide plus ADT group had a significantly longer OS [104]. In the LATITUDE trial, in which 1199 patients with mCSPC were randomized to abiraterone acetate plus prednisone (*n* = 597) or placebo (*n* = 602), the abiraterone acetate plus prednisone group had significantly prolonged OS compared with the placebo group (53.3 vs. 36.5 months, *p* < 0.0001, HR = 0.66) [105]. In the TITAN trial, in which 1052 patients with mCSPC were randomized 1:1 to apalutamide plus ADT or placebo plus ADT, the apalutamide plus ADT group had a significantly longer OS than the placebo group (not reached vs. 52.2 months, *p* < 0.0001, HR = 0.65) [106].

As mentioned above, ARST for mCSPC is effective. However, evidence on ARST for nmCSPC is limited. In the STAMPEDE trial, which randomized 1974 patients with high-risk nmCSPC to ARST plus ADT (*n* = 986) or ADT (*n* = 988), both groups did not reach the median OS; however, the ARST plus ADT group had a significantly longer OS (*p* < 0.0001, HR = 0. 60) [107]. Of the 1974 patients in this study, 774 (39%) had positive lymph nodes and 1684 (85%) received concomitant RT.

There are no reports of ARST being given for nmCSPC rather than in combination with other therapies. At present, ARST for nmCSPC is not recommended.

### 2.4. Adverse Effects of ADT in Older Patients

As discussed in the previous section, there are a variety of AEs in ADT. In particular, dementia, osteoporosis, and CV toxicity are important side effects of ADT in older patients. We summarize these AEs for older patients with PC.

#### 2.4.1. Risk of Developing Dementia Due to ADT in Older Patients

Low testosterone levels are associated with dementia risk in older men [108]. Low testosterone level and ADT have been reported to be associated with elevated levels of beta-amyloid protein, which characterizes Alzheimer’s disease [55,109]. As shown in Table 4, the association of ADT with dementia risk was reported in a large cohort study.

A retrospective cohort study of 100,414 older patients with PC, using data from the National Cancer Institute’s SEER program and Medicare, noted a 17% higher risk of dementia and 23% higher risk of Alzheimer’s disease in the ADT group [56].

A retrospective cohort study of 154,089 older patients with PC using the National Cancer Institute’s SEER-Medicare linked database reported a 14% increase in Alzheimer’s disease and 20% increase in dementia in the ADT group [57].

Based on data from the Prostate Cancer Database Sweden (PCBaSe Sweden), a retrospective cohort study of 146,985 men (with PC, *n* = 25,967; without PC, *n* = 121,018) was conducted [58]. Approximately 90% of the patients were older. The GnRH agonist PC group had a 15% increased risk of dementia compared with controls without PC.

However, a retrospective cohort study of 47,384 patients with PC from the National Health Insurance database in Taiwan and the Health Improvement Network in the United Kingdom (UK) showed contradictory results [59]. Approximately 70% of the patients were older. The incidence of dementia in the ADT group was 2.74 per 1000 person-years compared with 3.03 per 1000 person-years in the non-ADT group in Taiwan and 2.81 per 1000 person-years compared with 2.79 per 1000 person-years in the UK, with no significant difference between the ADT and non-ADT groups.

Two meta-analyses have examined the relationship between ADT and dementia risk. The first meta-analysis, including 50,541 patients with PC, showed a 47% increased risk of dementia in the ADT group [60]. The second meta-analysis, which included 339,400 cases treated with ADT and 436,851 controls, found a 21% increased risk of dementia in the ADT group [61].

Although some negative results were observed, the results of retrospective studies have suggested that ADT is causally associated with an increased risk of dementia. Further evidence from prospective studies is needed. The risk of dementia should be considered when providing ADT older patients with PC. Special consideration should be given to the risk of dementia in men at high-risk for cognitive decline.

#### 2.4.2. Risk of ADT-Induced Dementia in Older Patients

ADT causes deficiencies in testosterone and estrogen. Furthermore, it has been reported to decrease bone density by increasing bone turnover and resorption [110,111].

As shown in Table 5, several large retrospective cohort studies have investigated the increased risk of osteoporosis and fracture with ADT.

In a retrospective cohort study of 11,661 older patients with PC using medical claims data from a 5% national random sample of Medicare beneficiaries, the risk of fracture was 21% higher in the ADT group [62].

In a retrospective cohort study of 38,158 older patients with PC using linked administrative databases in Ontario, Canada, the risk of fracture was 46% higher in the ADT group [63].

Two large backward-looking cohort studies used the US National Cancer Institute’s SEER program and Medicare databases [64,65]. In the first retrospective cohort study of 50,613 older patients with PC between 1992 and 1997, the risk of fracture was 44% higher in the ADT group [64]. In the second retrospective cohort study of 80,844 older patients with PC between 1996 and 2003, the risk of fracture was 34% higher in the group of patients with non-metastatic PC who received ADT [65].

No large prospective studies have been performed to date, but a meta-analysis of a few cases was conducted. A meta-analysis of a prospective cohort study including 533 patients with PC found that bone mineral density (BMD) was significantly decreased in the ADT group [66].

Despite the paucity of prospective studies, previous ones have consistently shown that ADT reduces bone density and increases the risk of fracture. When ADT is performed in older patients, care must be taken to avoid fractures.

#### 2.4.3. Risk of CV Toxicity Due to ADT in Older Patients

In a retrospective cohort study of 73,196 older patients with localized PC using the SEER database, Keating et al. first showed in 2006 that GnRH agonist use significantly increased the risk of developing coronary artery disease, myocardial infarction, and sudden cardiac death [74].

Since then, various studies have investigated the association between ADT and CV risk events in patients with PC (Table 6).

In a retrospective cohort study of 37,443 patients (older patients accounted for a 60%) with local or regional PC diagnosed by the Veterans Healthcare Administration, CV toxicity was significantly higher in the ADT group [68].

Two large retrospective cohort studies using PCBaSe Sweden have reported CV toxicity [75,76]: (i) A retrospective cohort study of 41,362 patients with PC and 187,785 age-matched controls without PC. Older patients accounted for 90% of the study population. The risk of ischemic heart disease was 21% higher in the group using GnRH agonists [75]. (ii) Another retrospective cohort study of 42,263 patients with PC and 190,930 age-matched controls without PC. Older patients accounted for about 90% of the study population. The risk of thromboembolism was 67% higher in the group using GnRH agonists and 61% higher in the group that underwent surgical castration [76].

Several meta-analyses have also been conducted. A meta-analysis of retrospective cohort studies included 129,802 patients with PC who underwent ADT and 165,605 PC patients who did not undergo ADT. The results showed that the risk of CV disease (CVD) was 19% higher in the group using GnRH agonists than in the control group, and 46% higher in the CAB group. The risk of CV mortality was 17% higher in the ADT group [77].

A meta-analysis of both RCTs and observational studies included 74,538 patients with PC who received ADT and 85,947 patients without PC who did not receive ADT. The results showed 12% higher incidence of stroke in the ADT group than in the control group [78].

As mentioned above, many studies have shown that ADT increases the CV risk in patients with PC. However, some reports show no increased risk. A retrospective cohort study of 38,158 older patients with PC using linked administrative data at the Institute for Clinical Evaluative Sciences in Ontario, Canada, found no increased risk of acute myocardial infarction or sudden cardiac death in the ADT group [79].

Large cohort studies and meta-analyses have consistently reported an increased CV risk with ADT, and this complication should be considered with caution when providing ADT in older patients.

### 2.5. ADT in the Older

Results of retrospective studies have suggested that ADT alone may be indicated for high-risk localized and locally advanced PC in Asians, especially Japanese [58,59]. ADT alone is also an option for older patients with high-risk localized and locally advanced PC, regardless of ethnicity [15].

However, when ADT is used in older patients, side effects such as dementia, osteoporosis, and CV toxicity should be addressed. A meta-analysis of people aged ≥65 years revealed that patients with dementia had a significantly increased mortality risk compared with controls (odds ratio (OR) 2.63, 95% CI 2.17–3.21) [112].

The Lancet Commission on dementia prevention, intervention, and care identified 12 risk factors (low education, hypertension, hearing impairment, smoking, obesity, depression, physical inactivity, diabetes, infrequent social contact, alcoholism, head injury, and air pollution) for dementia that can be improved [113]. Improving these risk factors when administering ADT to older patients may help reduce the risk of developing dementia.

Fractures can be life-threatening in older patients receiving ADT. However, bisphosphonates and human monoclonal antibody (denosumab) can reduce the rate of bone loss in patients on ADT [114].

Moreover, a meta-analysis of 1824 patients with osteoporosis from 20 RCTs showed that kinesiology significantly improved BMD at the lumbar spine and femoral neck [115]. These preventive measures can reduce the risk of osteoporosis development in older patients, and ADT can be performed relatively safely.

CV toxicity is a life-threatening complication for older patients. A joint scientific statement in 2010 (American Heart Association, American Cancer Society, American Urological Association) has recommended assessment of CV profile before ADT initiation [116].

Metabolic syndrome (MetS) has been pointed out as a mechanism of CV toxicity caused by ADT [117]. Because testosterone maintains lean body mass, ADT-induced gonadal hypofunction was suggested to contribute to MetS development [118]. MetS is a collection of metabolic abnormalities including hypertension, central obesity, insulin resistance, and atherosclerotic dyslipidemia, and is considered an important CV risk factor [119]. A recent meta-analysis indicated that MetS doubles the CVD risk and increases all-cause mortality by 1.5 times [120].

Weight control is an important factor in MetS prevention [121]. For obese patients, a weight loss of 5–10 kg, even if not to normal weight, was shown to be effective in improving MetS and CV risk and increasing life expectancy [122]. Furthermore, losing at least 5% of body weight can lead to short-term improvements in insulin resistance, MetS, and related risk factors. In addition, a certain degree of aerobic exercise and physical activity has been found to contribute to CV risk reduction [123] and is recommended by the World Health Organization (WHO) 2020 guidelines on physical activity and sedentary behavior [124].

PDE5 inhibitors are gaining attention as agents to prevent increased CV risk [125]. A meta-analysis of randomized, placebo-controlled trials indicated that PDE5 inhibitors had anti-remodeling properties and improved cardiac inotropism with a good safety profile [126]. However, at this time, no studies have examined the efficacy of PDE5 inhibitors in reducing CV risk in patients with PC receiving GnRH agonists.

GnRH antagonists may also contribute to CV risk reduction. In a pooled analysis of six phase III prospective trials of 2328 patients with PC, patients using GnRH antagonists had a 56% reduction in cardiac events compared with patients using GnRH agonists [127].

A recent multinational randomized phase III trial reported that GnRH antagonist therapy, compared with a GnRH agonist, reduced adverse CV events by 54% in a total of 930 patients with advanced PC (GnRH agonist group, *n* = 308; GnRH antagonist group, *n* = 622) [128].

An RCT that investigated CVD-related mortality after treatment of advanced PC with atherosclerotic CVD with GnRH agonist or GnRH antagonist is currently underway and its results are awaited [129].

Further large-scale prospective studies are awaited, but administration of a GnRH antagonist rather than a GnRH agonist may prevent increased CV risk.

Other drugs besides ADT, such as angiogenesis inhibitors and immune checkpoint inhibitors, have also been reported, and future studies are awaited [130].

## 3. Conclusions

ADT alone for high-risk localized and locally advanced PC, while useful, is not generally first-line therapy. However, ADT may be a useful option for Asians, including Japanese and older patients, with measures to prevent adverse effects. We look forward to further research on racial differences in the efficacy of ADT and progress in countermeasures against adverse effects.

## Figures and Tables

**Table 1:** Definition of high-risk prostate cancer.

	Risk	Clinical Stage		Initial PSA		Gleason Score			References
D’Amico et al.	High	≥T2c	or	>20 ng/mL	or	≥8			[14]
NCCN 2021	High	T3a	or	>20 ng/mL	or	Grade Group 4 or Grade Group 5			[15]
	Very high	T3b/T4	or		or	Primary Gleason pattern 5 or > 4 cores with Grade Group 4 or 5	or	2 or 3 high-risk features	
EAU 2020	High	T2c	or	>20 ng/mL	or	≥8			[16]
	Locally advanced	T3/T4 or N1	and	Any	and	Any			
ESMO 2020	High	≥T2c	or	>20 ng/mL	or	≥8			[17]

PSA = Prostate specific antigen; NCCN = National Comprehensive Cancer Network; EAU = European Association of Urology; ESMO = European Society for Medical Oncology.

**Table 2:** Negative data of ADT for high-risk localized and locally advanced prostate cancer.

Study	Study Specification	Patient Characteristics	Size	Findings	References
Merglen et al. (2007)	retrospective cohort study	Patients with localized PC treated with either total prostatectomy, radiation therapy, watchful waiting, hormone therapy, or other treatment	844	Patients who received ADT alone already had an increased risk of PCSM at 5 years (HR 3.5, 95% CI 1.4–8.7)	[83]
Lee et al. (2018)	retrospective study	Patients diagnosed with localized PC who underwent ADT or treatment-free follow-up	340	In clinically unfavorable localized intermediate- and high-risk PC, initiation of ADT within 12 months of diagnosis was not associated with improved 5-year all-cause mortality or PCSM compared with patients who received no conservative treatment	[84]
Lu-Yao et al. (2008)	retrospective cohort study	Patients diagnosed with localized PC who underwent ADT or treatment-free follow-up	19,271	ADT is not associated with improved survival among the majority of elderly men with localized prostate cancer when compared with conservative management	[85]
Potosky et al. (2014)	retrospective cohort study	Newly diagnosed patients with localized PC	15,170	ADT was associated with neither a risk of all-cause mortality (HR 1.04, 95% CI 0.97–1.11) nor PCSM (HR 1.03, 95% CI 0.89–1.19).	[86]
Lu-Yao et al. (2014)	retrospective cohort study	Patients aged 66 years or older with localized PC who did not receive curative treatment	66,717	ADT is not associated with improved long-term overall or disease-specific survival for men with localized PC.	[87]
Sammon et al. (2015)	retrospective cohort study	Newly diagnosed patients with locally advanced or localized PC	46,376	There was an increased risk of all-cause mortality in the ADT group compared to the observation group (HR 1.37, 95% CI 1.20–1.56)	[82]

ADT = Androgen deprivation therapy; PC = Prostate cancer; PCSM = Prostate cancer specific mortality; HR = Hazard ratio; CI = Confidence interval.

**Table 3:** Positive data of ADT for high-risk localized and locally advanced prostate cancer.

Study	Study Specification	Patient Characteristics	Size	Findings	References
Labrie et al. (2002)	prospective study	Patients with newly diagnosed locally advanced or localized PC who have undergone CAB	57	In patients with stage T2–T3 cancer who continued CAB for more than 6.5 years and discontinued treatment there were only two cases of PSA elevation. Long-term continuous CAB was suggested to be a possibility for long-term control or cure of localized PC	[88]
Akaza et al. (2006)	prospective cohort study	Patients with newly diagnosed locally advanced or localized PC who have undergone ADT	151	In men with localized or locally advanced PC, primary ADT inhibited PC progression and resulted in a life expectancy similar to that of the normal population	[89]
Kawakami et al. (2006)	retrospective cohort study	Newly diagnosed localized PC patients with or without ADT	7044	The use of ADT therapy appeared to control disease in the majority of patients who received it, at least for an intermediate period	[90]
Akaza et al. (2010)	retrospective cohort study	Patients with newly diagnosed locally advanced or localized PC who have undergone ADT	15,461	ADT resulted in a long-term survival rate comparable to the general population	[91]
Matsumoto et al. (2014)	retrospective cohort study	Patients with newly diagnosed locally PC at intermediate to high risk who have undergone ADT	410	When prostate cancer with no capsular invasion and a GS of less than 8 was treated with ADT, the expected survival rate was similar to that of the general population	[92]
Studer et al. (2014)	randomized controlled trial	PC patients without distant metastasis treated with immediate or delayed ADT	985	Deferred ADT was inferior to immediate ADT in terms of overall survival (HR 1.21; 95% CI 1.05–1.39)	[93]
Nguyen et al. (2011)	meta-analysis of randomized controlled trial	Patients diagnosed with PC	4141	ADT was associated with lower PCSM (443/2527 vs. 552/2278 events; RR, 0.69; 95% CI, 0.56–0.84; *p* < 0.001) and lower all-cause mortality (1140/2527 vs. 1213/2278 events; RR, 0.86; 95% CI 0.80–0.93; *p* < 0.001)	[80]

ADT = Androgen deprivation therapy; PC = Prostate cancer; CAB = Combined androgen blockade; PSA = Prostate specific antigen; GS = Gleason score; HR = Hazard ratio; PCSM = Prostate cancer specific mortality; CI = Confidence interval; RR = Relative risk.

**Table 4:** Risk of ADT-induced dementia in older patients.

Study	Study Specification	Patient Characteristics	Size	Findings	References
Krasnova et al. (2020)	retrospective cohort study	Older patients diagnosed with PC who have received ADT or who have not received ADT	100,414	The risk of dementia was 17% higher and the risk of Alzheimer’s disease 23% higher in the group that received ADT	[56]
Jayadevappa et al. (2019)	retrospective cohort study	Older patients diagnosed with PC who have received ADT or who have not received ADT	154,089	Exposure to ADT, compared with no ADT exposure, was associated with a diagnosis of Alzheimer disease (HR 1.14, 95% CI, 1.10–1.18, *p* < 0.001) and dementia (HR 1.20, 95% CI 1.17–1.24, *p* < 0.001)	[57]
Robinson et al. (2019)	retrospective cohort study	Patients with PC and matched prostate-cancer-free controls (Older patients accounted for about 90%)	146,985	In men with prostate cancer, GnRH agonist treatment (HR 1.15, 95% CI 1.07–1.23) and orchiectomy (HR 1.60, 95% CI 1.32–1.93) were associated with an increased risk of dementia, as compared to no treatment in PC-free men	[58]
Liu et al. (2021)	retrospective cohort study	Patients diagnosed with PC who have received ADT or who have not received ADT (Older patients accounted for about 70%)	47,384	There was no statistical difference in the incidence of dementia between the ADT group and the group not receiving ADT (aHR, 1.12, 95% CI 0.87–1.43 in Taiwan, aHR 1.02, 95% CI: 0.85–1.23 in the UK)	[59]
Nead et al. (2017)	meta-analysis	Patients diagnosed with PC who have received ADT or who have not received ADT	50,541	ADT administration was associated with a 47% increase in dementia risk (HR 1.47, 95% CI 1.08–2.00, *p* = 0.02)	[60]
Cui et al. (2021)	meta-analysis	Patients diagnosed with PC who have received ADT or who have not received ADT	776,251	ADT administration was associated with a 21% increase in dementia risk (pooled HR = 1.21, 95% CI 1.13–1.30, *p* < 0.001)	[61]

PC = Prostate cancer; ADT = Androgen deprivation therapy; HR = Hazard ratio; CI = Confidence interval; GnRH = Gonadotropin releasing hormone; aHR = adjusted HR.

**Table 5:** Risk of developing osteoporosis due to ADT in older patients.

Study	Study Specification	Patient Characteristics	Size	Findings	References
Smith et al. (2005)	retrospective cohort study	Older patients diagnosed with PC who have received ADT or who have not received ADT	11,661	The rate of any clinical fracture was 7.88 per 100 person-years at risk in men receiving a GnRH agonist compared with 6.51 per 100 person-years in matched controls (RR 1.21, 95% CI, 1.14–1.29, *p* = 0.001)	[62]
Alibhai et al. (2010)	retrospective cohort study	Older patients diagnosed with PC who have received ADT or who have not received ADT	38,158	ADT was associated with an increased risk of fragility fracture (HR 1.65, 95% CI 1.53–1.78) and any fracture (HR 1.46, 95% CI 1.39–1.54)	[63]
Shahinian et al. (2005)	retrospective cohort study	Older patients diagnosed with PC who have received ADT or who have not received ADT	50,613	In patients who received ADT as primary treatment, the RR of any fracture was 1.44 (95% CI 1.33–1.56)	[64]
Beebe-Dimer et al. (2012)	retrospective cohort study	Older patients diagnosed with PC who have received ADT or who have not received ADT	80,844	ADT was associated with an increased rate of fracture in both non-metastatic patients (aHR 1.34, 95% CI 1.29–1.39) and metastatic patients (aHR 1.51, 95% CI 1.36–1.67)	[65]
Kim et al. (2019)	meta-analysis of prospective cohort study	Patients diagnosed with PC who have received ADT or who have not received ADT	533	Statistically significant decreases of BMD change relative to the control group were observed in the ADT treatment group in the lumbar spine (95% CI −6.72 to −0.47, *p* = 0.02), femoral neck (95% CI −4.73 to −1.48, *p* = 0.0002), and total hip (95% CI −2.99 to −0.19, *p* = 0.03)	[66]

PC = Prostate cancer; ADT = Androgen deprivation therapy; GnRH = Gonadotropin releasing hormone; RR = Relative risk; HR = Hazard ratio; CI = Confidence interval; aHR = adjusted HR; BMD = Bone mineral density; MD = Mineral density.

**Table 6:** Risk of cardiovascular toxicity due to ADT in older patients.

Study	Study Specification	Patient Characteristics	Size	Findings	References
Keating et al. (2006)	retrospective cohort study	Older patients with localized PC	73,196	GnRH agonist use was associated with increased risk of coronary heart disease (aHR 1.16, *p* < 0.001), myocardial infarction (adjusted HR 1.11, *p* = 0.03), and sudden cardiac death (aHR 1.16, *p* = 0.004)	[74]
Keating et al. (2010)	retrospective cohort study	Patients diagnosed with local or regional PC (older patients accounted for about 60%)	37,443	The group of patients who received ADT was significantly more likely to have coronary artery disease (aHR 1.19, 95% CI = 1.10–1.28), myocardial infarction (aHR 1.28, 95% CI = 1.08–1.52), sudden cardiac death (aHR 1.35, 95% CI = 1.18–1.54), and stroke (aHR 1.22, 95% CI = 1.10–1.36) were increased	[68]
O’Farrell et al. (2015)	retrospective cohort study	Patients with PC and matched PC-free controls (older patients accounted for about 90%)	229,147	CVD risk was increased in men on GnRH agonists compared with the comparison cohort (HR 1.21, 95% CI 1.18–1.25)	[75]
O’Farrell et al. (2016)	retrospective cohort study	Patients with PC and matched PC-free controls (older patients accounted for about 90%)	233,193	GnRH agonist users and surgically castrated men had a higher risk of thromboembolic disease than the comparison cohort: HR 1.67, 95% CI 1.40–1.98 and HR 1.61, 95% CI 1.15–2.28, respectively	[76]
Zhao et al. (2014)	meta-analysis of retrospective cohort study	Patients diagnosed with PC who have received ADT or who have not received ADT	295,407	CVD was related to GnRH (HR 1.19, 95% CI 1.04–1.36, *p* < 0.001) and GnRH plus oral antiandrogen (HR 1.46, 95% CI 1.03–2.08, *p* = 0.04). ADT was associated with cardiovascular mortality (HR 1.17, 95% CI 1.04–1.32, *p* = 0.01)	[77]
Meng et al. (2016)	meta-analysis of retrospective cohort study	Patients diagnosed with PC who have received ADT or who have not received ADT	160,485	The incidence of stroke in ADT users was 12% higher than control groups, (HR 1.12, 95% CI 0.95–1.32, *p* = 0.16)	[78]
Alibhai et al. (2009)	retrospective cohort study	Older patients diagnosed with prostate cancer who received ADT or who were not diagnosed with prostate cancer who did not receive ADT	38,158	ADT use was not associated with AMI (HR 0.91, 95% CI 0.84–1.00) or sudden cardiac death (HR 0.96, 95% CI 0.83–1.10)	[79]

PC = Prostate cancer; GnRH = Gonadotropin releasing hormone; HR = Hazard ratio; aHR = adjusted HR; CI = Confidence interval; ADT = Androgen deprivation therapy; CVD = cardiovascular disease; CAB = Combined androgen blockade.
